# *In vivo* antioxidant activity of mackerel (*Scomber japonicus*) muscle protein hydrolysate

**DOI:** 10.7717/peerj.6181

**Published:** 2018-12-21

**Authors:** Khawaja Muhammad Imran Bashir, Md. Mohibbullah, Jeong Hyeon An, Ji-Yeon Choi, Yong-Ki Hong, Jae Hak Sohn, Jin-Soo Kim, Jae-Suk Choi

**Affiliations:** 1 Seafood Research Center, IACF, Silla University, Busan, Republic of Korea; 2 Research Center for Extremophiles and Microbiology, College of Medical and Life Sciences, Silla University, Busan, Republic of Korea; 3 Department of Fisheries and Marine Bioscience, Bangabandhu Sheikh Mujibur Rahman Science and Technology University, Gopalgonj, Bangladesh; 4 Southeast Medi-Chem Institute, Busan, Republic of Korea; 5 Department of Biotechnology, College of Fisheries Sciences, Pukyong National University, Busan, Republic of Korea; 6 Department of Food Biotechnology, Division of Bioindustry, College of Medical and Life Sciences, Silla University, Busan, Republic of Korea; 7 Department of Seafood and Aquaculture Science, Gyeongsang National University, Tongyeong-si, Gyeongsangnam-do, Republic of Korea

**Keywords:** SOD and CAT protein expression, *In vivo* antioxidant activity, Protamex, Enzymatic hydrolysis, Protein Hydrolysate

## Abstract

Pacific chub mackerel (*Scomber japonicus*) is an important fish throughout the world, especially in East Asian countries, including Korea, China, and Japan. Protein hydrolysates from marine sources are commonly used as nutritional supplements, functional ingredients, and flavor enhancers in the food, beverage, and pharmaceutical industries. Antioxidants isolated from fish are relatively easy to prepare, are cost effective, and have no reported side effects. Hence, the present study aimed to investigate the *in vivo* antioxidant activities of mackerel muscle protein hydrolysate (MMPH) prepared using Protamex. The *in vivo* bioactivities of MMPH were investigated in alcoholic fatty liver mice (C57BL/6). Serum alanine aminotransferase and aspartate aminotransferase levels were comparable in test and control mice, whereas serum triglyceride and lipid peroxidation levels significantly (*p* < 0.05; *p* < 0.001) decreased after administration of MMPH (100–500 mg kg^−1^), especially at a concentration of 100 mg kg^−1^. A significant (*p* < 0.05) reduction in xanthine oxidase activity was observed in all groups treated with MMPH (100–500 mg kg^−1^), as compared with the control group. Significantly (*p* < 0.05) higher superoxide dismutase (SOD) activity/protein expression and regulated catalase (CAT) activity/protein expression levels were observed in groups administered MMPH (100–500 mg kg^−1^), especially at a concentration of 100 mg kg^−1^. These results show that the abundant amino acids of *S. japonicus* play an important role in the cytosol of the liver cells by directly participating in the expression of xanthine oxidase and the detoxifying SOD and CAT proteins, thereby enhancing antioxidant ability and ultimately, inhibiting lipid peroxidation. This study demonstrated that muscle protein hydrolysate from *S. japonicus* has strong antioxidant activities.

## Introduction

Antioxidants are biomolecules that are associated with preventing or slowing down oxidative damage in the body. Aerobic metabolism generates reactive oxygen species (ROS; Kumar, Nazeer & Jaiganesh, 2012), which are responsible for oxidative damage. ROS are oxygen derivatives with a highly reactive and unstable electron in their outermost orbit, which enables them to react with biological macromolecules, such as lipids, proteins, and DNA (Schieber & Chandel, 2014; Nicco & Batteux, 2018). The oxidative stress regenerated by ROS can be controlled by antioxidant defense mechanisms, including endogenous enzymes, natural antioxidants, and dietary supplements. Antioxidant activity is generally assessed by determining the scavenging activity of free radicals and ROS through *in vitro* and *in vivo* assays, which assess 1,1-diphenyl-2-picrylhydrazyl (DPPH)-radical scavenging activity, oxygen-radical absorbance capacity, 2,2′-azino-bis 3-ethylbenzothiazoline-6-sulfonic acid diammonium salt (ABTS)-radical scavenging activity, hydroxyl-radical scavenging activity, superoxide anion-radical scavenging activity, superoxide dismutase (SOD) expression, and catalase (CAT) enzyme expression (Bashir et al., 2018a).

Fish protein hydrolysates and peptides are extensively used as nutraceuticals, functional constituents, and nutritional supplements in the beverage, pharmaceutical, and food industries (Chalamaiah et al., 2012; Sheriff et al., 2014; Ishak & Sarbon, 2018). Dietary proteins containing biologically active peptides have been associated with antihypertensive, antithrombotic, antimicrobial, anticancer, antioxidant, and immunomodulatory effects (Pádraigín et al., 2012; Udenigwe & Aluko, 2012). The negative consumer perception of synthetic antioxidants, such as butylated hydroxyl toluene, butylated hydroxyl anisole, tert-butylhydroquinone, and n-propyl gallate, limit their application in the food industry (Kumar et al., 2015). However, peptides and protein hydrolysates purified from marine sources are considered a reliable source of antioxidants, with no reported adverse effects (Liu et al., 2016).

Several pharmaceutically significant biomaterials from fishery sources have been extracted and analyzed for their activity in *in vitro* and *in vivo* model assays. Protein hydrolysates and peptides derived from fishery sources, including Alaska pollock (Jia et al., 2010), sardine (Ali et al., 2010), horse mackerel (Kumar, Nazeer & Jaiganesh, 2011), sea urchin (Qin et al., 2011), seela and ribbon fish (Nazeer et al., 2011), croaker (Kumar, Nazeer & Jaiganesh, 2012), Pacific hake (Cheung et al., 2012), Pacific whiting (Mazorra-Manzano et al., 2012), tilapia (Fan et al., 2012), grass carp (Wang et al., 2013), giant squid (Mosquera et al., 2016), yellowfin tuna (Oliveira et al., 2017), and smooth-hound (Tao et al., 2018), have been reported to possess strong antioxidant properties.

*Scomber japonicus* (Pacific chub mackerel) is a mid-sized near-coastal fish species found at depths of 0 to 300 m in temperate regions in nearby seas of the northwest Indian and Pacific oceans (Bashir et al., 2018a). Worldwide *S. japonicus* catches of 1.8 million tons were documented in 2014 (FAO, 2014) *S. japonicus* muscle is believed to be a unique source of nutrients and palatable proteins, due to its excellent amino acid composition (Oduro, Choi & Ryu, 2011; Kumar, Nazeer & Jaiganesh, 2012; Sheriff et al., 2014). Mackerel is a well-known fish consumed in East Asian countries, including Korea, China, and Japan. It is considered an economically imperative fish in Korea (MOF, 2016). However, with the exception of a previous report by our group (Bashir et al., 2018a), there are no reports available on the preparation of protein hydrolysates by enzymatic hydrolysis of *S. japonicus*.

In our previous studies, we have reported on the *in vitro* bioactivity of protein hydrolysates from marine sources, prepared by enzymatic hydrolysis and sub-critical water hydrolysis (Choi et al., 2016; Choi et al., 2017; Bashir et al., 2018a). The aim of the current study was to investigate the *in vivo* antioxidant activities of muscle protein hydrolysates of Pacific chub mackerel, prepared by enzymatic hydrolysis.

## Material and Methods

### Preparation of mackerel muscle protein hydrolysate (MMPH)

*S. japonicus* was purchased from a retail shop in Busan, Republic of Korea. Fish muscle samples for enzymatic hydrolysis were prepared as described previously by Bashir et al. (2018a). Briefly, 100 g of muscle sample was mixed with 10 volumes of 0.1 M potassium phosphate buffer (pH 8) and homogenized thoroughly. The proteases, Alcalse, Neutrase, and Protamex (Novozymes, Bagsvaerd, Copenhagen, Denmark), were added to bioreactors separately, at 2% of the working volume of the sample. Bioreactors were incubated for 1 h in a shaking incubator (Vision Scientific, Daejeon, Rep. of Korea) at 110 rpm and 50 °C. Ten-milliliter samples were taken at 30 min intervals. Samples were first incubated at 100 °C for 15 min to inactivate the proteases and then, cooled at 4 °C. Proteins were separated by centrifugation at 4,000× g for 15 min and then transferred into new tubes. Hydrolyzed samples were filtered through 0.45 µm cellulose acetate filter disks (Agilent Technologies, Hachioji, Japan) and stored at −20 °C for later use. The degree of hydrolysis was calculated as described previously by Bashir et al. (2018a).

### *In vitro* bioactivity of muscle protein hydrolysate

Previously, we reported the *in vitro* antioxidant activity of *S. japonicus* muscle protein hydrolysates (Bashir et al., 2018a). In our previous report, hydrolysates prepared with a hydrolysis time <2 h showed significant (*p* < 0.05) antioxidant activities and higher antioxidant activity was seen in hydrolysates prepared by hydrolysis with Protamex for 1 h. Thus, only MMPHs prepared by Protamex hydrolysis were used in this study.

### *In vivo* antioxidant activity of mackerel muscle protein hydrolysate

#### Experimental animals

Experimental animals (6-week-old male C57BL/6 mice) were purchased from SamTaco BioKorea (Osan, Rep. of Korea). Animals were quarantined and acclimatized for 1 week at the Southern Institute of Animal Science, Busan, Rep. of Korea (Registration No. 412). Only healthy animals were used in these experiments. The breeding environment was set at a relative humidity of 50 ± 10%, a temperature of 22 ± 3 °C, and a 12 h (07:00 to 19:00) photoperiod. During the acclimatization period, a normal diet (AIN-93 diet) was supplied and drinking water was freely consumed. Animals were then assigned to 6 groups, comprising 8 animals each.

Experiments were performed according to the national policies and regulations of the usage and welfare of laboratory animals and were approved by the Institutional Animal Care and Use Committee of Southeast Medi-Chem Institute (SEMI), Busan, Rep. of Korea (Approval No. SEMI-17-01).

#### Dietary composition and sample administration

Animals were supplied with a Lieber-DeCarli (Diet Inc., Bethlehem, PA, USA) liquid diet (control diet) or an ethanol diet for 42 d. The nutrient constituents and calorie contents of the control and ethanol diets are shown in Table 1. Each experimental group consisted of 8 animals, with four animals per cage (Table 2). The liquid diet (20 mL per animal) was orally administered at a predetermined period for 6 weeks (Fengler et al., 2016). To avoid ethanol rejection and to set up the adaptation period, the ethanol concentration in the ethanol diet was increased by 0.5% from the initial 0.5% to a final concentration of 3.3% at the end of 2 d, according to the previously reported method of Bertola et al. (2013), with slight modifications.

**Table 1:** Composition of standard Lieber-DeCarli diet used in this study.

Ingredient	Control diet	Ethanol diet
Casein	176.778	176.778
L-cystine	2	0
DL-methionine	1.2	0
Corn oil	75.1	75.14
Olive oil	251.056	251.056
Safflower oil	23.868	23.868
Maltose dextrin	456.192	101.376
Cellulose	0	0
Mineral mix	4.1125	4.1125
Vitamin mix	9.5	9.5
Choline bitartrate	0	0
Xanthan gum	0	0
Ethanol	–	358.45

**Notes.**

Unit: kcal L^−1^; The basal diet was formulated and supplied from Diets Inc. (Bethlehem, PA, USA) according to the recommendations of the AIN.

**Figure 1:**
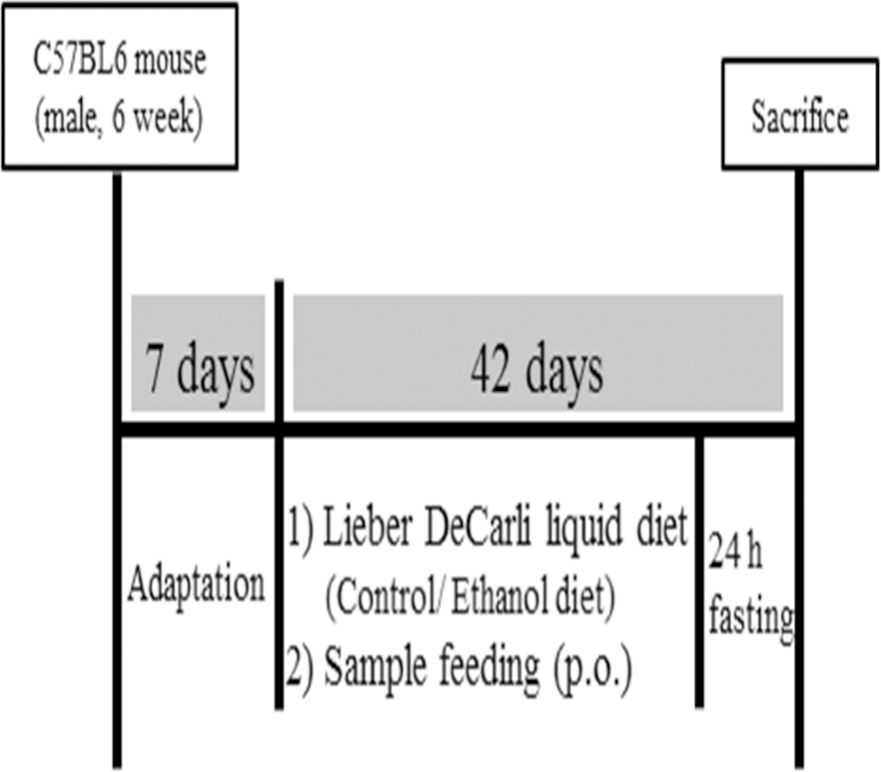
Animal experimental design.

Mice were divided into the following 6 experimental groups: normal (N group), negative control (C group), positive control (P group; silymarin 100 mg kg^−1^; Sigma-Aldrich Co., St. Louis, MO, USA), M100 (MMPH 100 mg kg^−1^), M250 (MMPH 250 mg kg^−1^), and M500 (MMPH 500 mg kg^−1^), as shown in Table 2. Silymarin, a well-known hepatoprotective extract purified from the milk thistle plant (*Silybum marianum*), containing many flavonolignans, was used as a positive control, based on the previously reported studies (Jian et al., 2010; Surai, 2015; Wang et al., 2016; Zhong et al., 2017) showing that it has no side effects on animals. Silymarin is frequently used in the treatment of liver disorders, as it protects liver cells by preventing lipid peroxidation and glutathione depletion and thus, stabilizing membrane permeability (Franschini, Demartini & Esposti, 2002; Jian et al., 2010; Zhong et al., 2017). Test materials, including controls, were administered orally, once a day for 42 d, at a dose of 10 mL kg^−1^ (Fengler et al., 2016), as shown in Fig. 1. Forty-two days after the administration of the test sample, animals fasted (only water was supplied) for 24 h and were subsequently sacrificed within a specified time period (10:00–12:00 am) to control for the fluctuation of enzyme activity.

**Table 2:** Experimental design of animals used in this study.

Sr. #	Group	Design		No. of mice
1	N	Liber-DeCarli control (Liquid dietary supplement)	Solvent control (D.W., p.o.)	8
2	C	Liber-DeCarli ethanol (Liquid dietary supplement)		8
3	P		Silymarin(100 mg kg^−1^ p.o.)	8
4	M100		MMPH(100 mg kg^−1^ p.o.)	8
5	M250		MMPH(250 mg kg^−1^ p.o.)	8
6	M500		MMPH(500 mg kg^−1^ p.o.)	8

**Notes.**

N normal C control P positive control DW distilled water MMPH mackerel muscle protein hydrolysate

#### Measurement of changes in body and organ weight

Body weight was measured twice a week. The Lieber-DeCarli liquid diet and experimental specimens were orally administered for 42 d, after which the animals fasted (only water supplied) for 24 h and dissected. Body and organ weights were measured with an electronic measuring balance (IGZ Instruments AG, Zurich, Switzerland). To account for variances due to individual body weight, relative organ/tissue weight (% of body weight) was calculated, according to a previously reported method (Lee et al., 2015).

#### Serum biochemical analysis

Experimental animals were anesthetized with CO_2_ and blood was collected from the abdominal aorta using a 1 mL syringe. Serum was prepared using a standard serum preparation method, as described by Yesufu et al. (2010). Briefly, blood from the abdominal aorta was collected in clotting-activated serum tubes, incubated at room temperature for 30 min to clot, and then centrifuged at 2,000× g for 15 min to obtain serum. The isolated serum was analyzed for alanine aminotransferase (ALT), aspartate aminotransferase (AST), total cholesterol, and triglyceride content using a serum biochemistry analysis kit (Hoffmann-La Roche AG, Basel, Switzerland). The remaining serum was stored in an ultra-low temperature freezer (Sanyo Trading Co. Ltd., Tokyo, Japan) at −150 °C.

#### Measurement of liver lipid peroxidation content

Liver tissue was treated with 0.1 M sodium phosphate buffer (pH 7.4) and, under ice-cold conditions, a 10% homogenate was prepared using a glass Teflon homogenizer. The resulting solution was regarded as a liver homogenate fraction. Tissue homogenates were stored in an ultra-low temperature freezer (Sanyo Trading Co. Ltd.) at −150 °C until further use.

Lipid peroxidation in liver tissue was measured by a modified thiobarbituric-acid-reactive substance fluorescence method (Reitznerová et al., 2017). Briefly, liver samples were deproteinized with 1 mL of 0.6% thiobarbituric acid and 1 mL of 14% trichloroacetic acid. The reaction was completed by incubating the mixture in a water bath for 30 min and then cooling on ice for 5 min. The absorbance of the colored product was measured at 532 nm with a UV/VIS spectrophotometer (Mecasys, Daejeon, Rep. of Korea). The final product of lipid peroxidation, 1,1,3,3-tetraethoxypropane, was used as a reference material. Results were expressed as nM malondialdehyde (MDA) generated from 1 g of liver tissue.

#### Measurement of protein content

Serum total protein content was measured by following the Bradford assay kit manufacturer’s instructions (Sigma-Aldrich). Briefly, 0.1 mL of serum was mixed with equal volumes of 0.1 M NaOH and vortexed for 1 min. Five milliliters of Bradford’s reagent was added to the mixture and it was incubated at room temperature for 5 min. The intensity of the developed blue color was measured at 595 nm with a spectrophotometer. Bovine serum albumin (BSA; Thermo Fisher Scientific Inc., Rockford, IL, USA) was used as a standard and protein levels were expressed as mg g^−1^ of tissue.

#### Antioxidant enzyme activity assays

Aldehyde oxidase (AO) and xanthine oxidase (XO) activities of liver tissues were measured using a mouse aldehyde oxidase ELIZA kit and a mouse xanthine oxidase ELIZA kit (Mybiosource Inc., San Diego, CA, USA), according to the manufacturer’s instructions. SOD enzyme activity was determined according to the previously reported method of Weydert and Cullen (Weydert & Cullen, 2010), with slight modifications. SOD activity in this assay is based on the xanthine-xanthine oxidase system generating a superoxide flux, with superoxide production being detected using nitroblue tetrazolium (NBT). SOD activity was measured spectrophotometrically at 560 nm. One unit was defined as the amount of enzyme providing a 50% inhibition of NBT reduction (Onoja et al., 2014). Results were expressed as U mL^−1^. CAT enzyme activity was estimated according to the method of Atawodi (2011), with slight modifications. CAT enzyme activity was defined as the amount of enzyme needed to decompose 1 nM of hydrogen peroxide (H_2_O_2_) in one min, at 25 °C and pH 7.8. The decomposition rate of H_2_O_2_ in the presence of CAT was measured spectrophotometrically at 240 nm.

#### SOD and CAT protein expression by immunoblotting

To measure protein expression, cells were lysed in cell lysis buffer, homogenized, then centrifuged at 11,000× g for 20 min at 4 °C to remove the supernatant. Total protein concentration was quantitated by Bradford assay (Sigma-Aldrich) and protein samples were analyzed by sodium dodecyl sulfate-polyacrylamide gel electrophoresis, as described previously by Bashir et al. (in press). Protein extracts were separated on 12% polyacrylamide gels and subsequently, transferred to polyvinylidene fluoride membranes using a semi-dry transfer system (Bio-Rad, Hercules, CA, USA). Membranes were washed twice with 1 × Tris-buffered saline (TBS) for 10 min each wash, incubated for 1 h in blocking solution (5% skim milk (Fujifilm Wako Chemicals, Richmond, VA, USA) and 1 × TBST buffer), and washed thrice with 1 × TBST for 10 min each wash. Membranes were separately incubated overnight at 4 °C with SOD monoclonal antibody (Santa Cruz Biotechnology Inc., Santa Cruz, CA, USA) or CAT monoclonal antibody (Santa Cruz Biotechnology), diluted to 1:1,000 in 5% skim milk. Membranes were then washed thrice with 1 × TBST for 10 min each wash and incubated for 1 h with goat anti-mouse IgG (H+L) HRP-conjugated secondary antibody (Thermo Fisher Scientific), diluted to 1:1,000 in 5% skim milk. Blots were then washed thrice with 1 × TBST for 10 min each wash. SOD and CAT expression was detected using a West Save Gold western blot detection kit (Abfrontier, Seoul, Rep. of Korea) and band intensity was measured using a Chemi-Doc XRS system (Bio-Rad). Results were normalized using a *β*-actin monoclonal antibody (Santa Cruz Biotechnology) as an internal standard.

#### Statistical analysis

Experimental data were expressed as means ± standard deviation (S.D.) or standard error (S.E.) of 8 animals. Data were analyzed using the Statview statistical program (SAS Institute Inc., Cary, NC, USA). Statistical significance was verified by one-way analysis of variance (ANOVA) and results were considered statistically significant at *p* < 0.05, *p* < 0.01, and *p* < 0.001.

## Results

### Changes in body, organ, and tissue weight

Body weight of experimental animals was measured at intervals of 7 days from the time of acclimatization until 6 weeks after sample administration. Both the control and experimental groups showed significantly (*p* < 0.05) consistent weight gain over this period (Table 3). However, groups C, P, M100, M250, and M500 fed the Lieber-DeCarli ethanol diet did not show a significant (*p* < 0.05) increase in body weight compared to group N. Body weight in the intact control group changed from 20.41 ± 1.66 g to 30.26 ± 2.64 g; in group C, from 19.68 ± 1.84 g to 27.85 ± 0.70 g; and in group P, from 20.01 ± 0.82 g to 27.09 ± 1.22 g. Body weight in groups administered MMPH (100, 250, and 500 mg kg^−1^) changed from 20.49 ± 1.79 g to a maximum of 26.99 ± 2.37 g in the M500 group.

**Table 3:** Effect of mackerel muscle protein hydrolysate on body weight gains.

Sr. #	Group	Body weight (g)						
		**Initial**	**1 week**	**2 week**	**3 week**	**4 week**	**5 week**	**6 week**
1	N	20.41 ± 1.66^n.s^	21.86 ± 1.01	22.96 ± 1.56	25.91 ± 2.17	26.44 ± 12.50	28.69 ± 2.26	30.26 ± 2.64^*^^,^^e^
2	C	19.68 ± 1.84	22.09 ± 0.98	22.89 ± 1.04	24.79 ± 1.02	25.25 ± 0.76	26.99 ± 1.02	27.85 ± 0.70
3	P	20.01 ± 0.82	21.21 ± 0.68	22.24 ± 1.11	23.39 ± 1.29	23.91 ± 1.34	25.69 ± 1.24	27.09 ± 1.22
4	M100	20.64 ± 0.87	21.19 ± 1.29	21.91 ± 1.91	22.96 ± 1.39^*^^,^^c^	23.63 ± 1.37	2509 ± 1.58	25.88 ± 1.64^*^^,^^f^
5	M250	20.49 ± 1.79	21.01 ± 0.74^*^^,^^a^	21.46 ± 1.79^*^^,^^b^	23.38 ± 1.58	23.99 ± 1.83	24.59 ± 3.36^*^^,^^d^	26.94 ± 1.98
6	M500	20.55 ± 1.46	21.11 ± 1.39	22.26 ± 1.47	23.79 ± 1.75	24.46 ± 1.70	26.35 ± 2.29	26.99 ± 2.37

**Notes.**

Values are expressed as mean ± S.D. for groups of eight animals.

N normal C control P positive control M100, M250, and M500 mackerel muscle protein hydrolysate at concentrations of 100, 250, and 500 mg kg^−1^, respectively. n.s not significant

* *P* < 0.05 vs. control.

(a–f) represent the significance level of *p* for ANOVA calculated by significance between control and test groups using Fisher’s PLSD post hoc test. *a* = 0.0467; *b* = 0.0460; *c* = 0.0255; *d* = 0.0280; *e* = 0.0012; *f* = 0.0412.

Significantly (*p* < 0.05) higher liver (1.10 ± 0.14 g) and epididymal white adipose tissue (1.20 ± 0.16 g) weights were observed in group N compared to the other groups. However, there were no significant differences in kidney, spleen, heart, testis, or retroperitoneal white adipose tissue weight (Table 4). The relative weights of liver and epididymal white adipose tissue in mice administered the Lieber-DeCarli ethanol diet for 6 weeks did not change with different MMPH concentrations. However, the M100 group showed a reduced kidney weight (0.33 ± 0.05 g) compared to the controls (Table 5).

**Table 4:** Effect of mackerel muscle protein hydrolysate on organ and tissue weight.

Sr. #	Group	Liver	Kidney	Spleen	Heart	Testis	Retroperitoneal white adipose tissue	Epididymal white adipose tissue
1	N	1.10 ± 0.14^*^^,^^a^	0.37 ± 0.05^n.s^	0.11 ± 0.04^n.s^	0.14 ± 0.05^n.s^	0.25 ± 0.08^n.s^	0.36 ± 0.06^n.s^	1.20 ± 0.16^*^^,^^c^
2	C	0.96 ± 0.05	0.41 ± 0.04	0.10 ± 0.00	0.13 ± 0.05	0.20 ± 0.05	0.24 ± 0.04	0.91 ± 0.27
3	P	0.94 ± 0.12	0.39 ± 0.06	0.13 ± 0.06	0.15 ± 0.05	0.19 ± 0.10	0.19 ± 0.04	0.91 ± 0.37
4	M100	0.88 ± 0.15	0.33 ± 0.05^*^^,^^b^	0.10 ± 0.10	0.13 ± 0.05	0.23 ± 0.07	0.23 ± 0.05	0.86 ± 0.21
5	M250	0.94 ± 0.11	0.44 ± 0.16	0.10 ± 0.00	0.13 ± 0.05	0.20 ± 0.05	0.21 ± 0.07	0.81 ± 0.39
6	M500	0.90 ± 0.05	0.35 ± 0.05	0.10 ± 0.00	0.14 ± 0.05	0.21 ± 0.06	0.34 ± 0.04	0.91 ± 0.16

**Notes.**

Unit: g; values are expressed as mean ± S.D. for groups of eight animals; N: normal; C: control; P: positive control; M100, M250, and M500: mackerel muscle protein hydrolysate at concentrations of 100, 250, and 500 mg kg^−1^, respectively. n.s: not significant; *p < 0.05 vs. control. ^a−c^ represent the significance level of *p* for ANOVA calculated by significance between control and test groups using Fisher’s PLSD post hoc test. *a* = 0.0167; *b* = 0.0335; *c* = 0.0432.

**Table 5:** Changes in relative liver and epididymal fat weight.

Sr. #	Group	Relative liver weight	Relative epididymal white adipose tissue weight
1	N	0.036 ± 0.005^n.s^	0.040 ± 0.005^n.s^
2	C	0.035 ± 0.005	0.033 ± 0.009
3	P	0.033 ± 0.005	0.031 ± 0.012
4	M100	0.034 ± 0.005	0.033 ± 0.009
5	M250	0.034 ± 0.005	0.036 ± 0.008
6	M500	0.031 ± 0.004	0.033 ± 0.005

**Notes.**

Unit: g g^−1^ of body weight; values are expressed as mean ± S.D. for groups of eight animals; N: normal; C: control; P: positive control; M100, M250, and M500: mackerel muscle protein hydrolysate at concentrations of 100, 250, and 500 mg kg^−1^, respectively. n.s: not significant.

### Serum biochemical analysis

**Figure 2:**
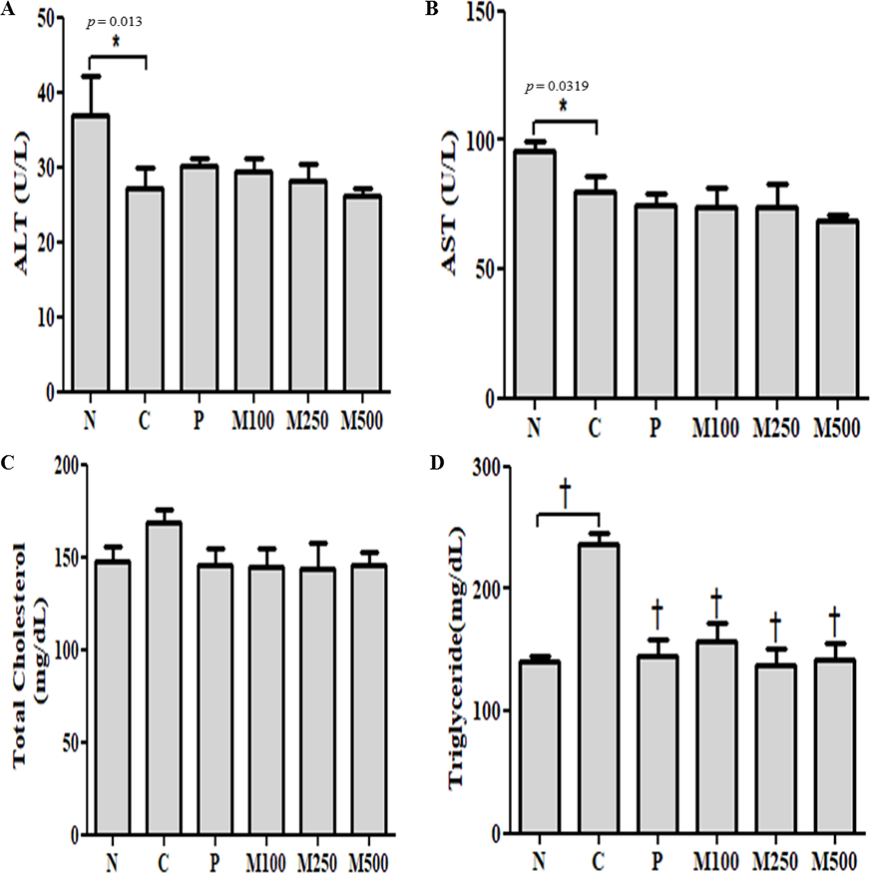
Serum biochemical properties. Serum (A) ALT; (B) AST; (C) total cholesterol; and (D) triglyceride levelsValues are expressed as mean ± S.E. for groups of eight animals; N: normal; C: control; P: positive control; M100, M250, and M500: mackerel muscle protein hydrolysate at concentrations of 100, 250, and 500 mg kg^−1^, respectively. n.s: not significant; ∗*p* < 0.05 vs. control, †*p* < 0.001 vs. control *p: p* value of ANOVA and significance between control and test groups using Fisher’s PLSD post hoc test.

A significant (*p* < 0.01) decrease in the serum ALT and AST levels and a significant (*p* < 0.01) increase in serum total cholesterol and triglyceride levels were observed in the ethanol-fed group C, compared to group N. However, serum ALT, AST, total cholesterol and triglyceride levels were significantly (*p* < 0.001) lower in all MMPH treatment groups (M100, M250, and M500), compared to the control groups (group N, P, and C; Fig. 2).

Serum ALT levels in group C and group P were 27.24 ± 6.17 and 30.17 ± 2.95 U L^−1^, respectively. Serum ALT levels in the MMPH-fed groups, M100, M250, and M500, were 29.40 ±  4.86, 28.15 ± 5.67, and 26. 21 ± 2.79 U L^−1^, respectively, showing significantly (*p* < 0.05) lower values than the group N (36.98 ± 10.61 U L^−1^). Serum AST levels in the group C and group P were 80.25 ±  14.04 and 74.96 ± 11.07 U L^−1^, respectively. Serum AST levels in the MMPH-fed groups, M100, M250, and M500, were 73.65 ± 21.19, 74.19 ± 23.26, and 68.53 ± 6.87 U L^−1^, respectively, which were lower than in group N (95.88 ± 8.35 U L^−1^).

Total cholesterol concentration was 168.21 ± 18.36 mg dL^−1^ in group C fed the Lieber-DeCarli ethanol diet, compared to 147.85 ± 16.05 mg dL^−1^ in group N. In contrast, total cholesterol concentration decreased to 144.39  ± 27.84 mg dL^−1^, 144.10 ± 33.97 mg dL^−1^, and 145.28 ± 19.11 mg dL^−1^ after oral administration of MMPH (M100, M250, and M500, respectively) for 6 weeks (Fig. 2C).

Triglyceride concentration, which is directly involved in lipid metabolism, significantly (*p* < 0.001) increased to 236.32 ± 20.01 mg dL^−1^ in group C fed the Lieber-DeCarli ethanol diet for 6 weeks, compared to 140.07 ± 10.67 mg dL^−1^ in group N (Fig. 2D). However, triglyceride concentration decreased significantly (*p* < 0.001) in group P (145.48 ± 36.03) and MMPH groups, M100 (157.44 ± 39.08 mg dL^−1^), M250 (137.16 ± 33.89 mg dL^−1^), and M500 (142.40 ± 35.77 mg dL^−1^).

### Measurement of lipid peroxidation content

**Figure 3:**
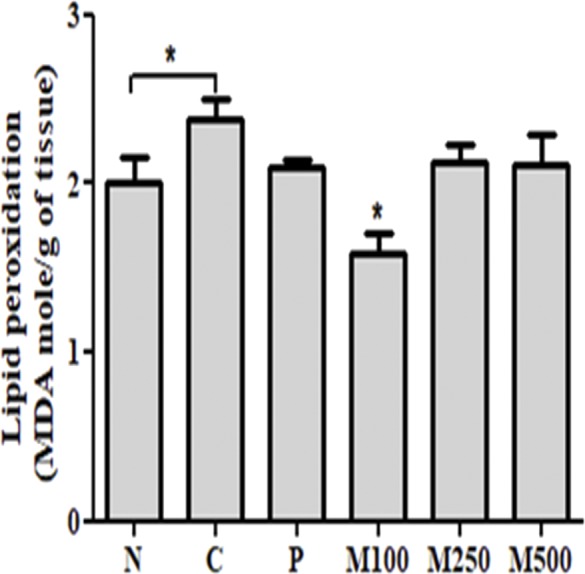
Effect of mackerel muscle protein hydrolysate on liver lipid peroxidation levels. Values are expressed as mean ± S.E. for groups of eight animals; N: normal; C: control; P: positive control; M100, M250, and M500: mackerel muscle protein hydrolysate at concentrations of 100, 250, and 500 mg kg^−1^, respectively. n.s: not significant; ∗*p* < 0.05 vs. control *p: p* value of ANOVA and significance between control and test groups using Fisher’s PLSD post hoc test.

Lipid peroxidation in liver tissue from group C animals fed the Lieber-DeCarli ethanol diet (2.38 ± 0.29 MDA nmol g^−1^) increased significantly (*p* < 0.05) compared to group N (2.00 ± 0.36 MDA nmol g^−1^; Fig. 3). A significantly (*p* < 0.05) reduced lipid peroxidation level was observed for group M100 (1.58 ± 0.29 MDA nmol g^−1^). Lipid peroxidation levels in the other MMPH groups (M250 and M500) were not significantly different from the levels in group P.

### Aldehyde oxidase and xanthine oxidase enzyme activity

**Figure 4:**
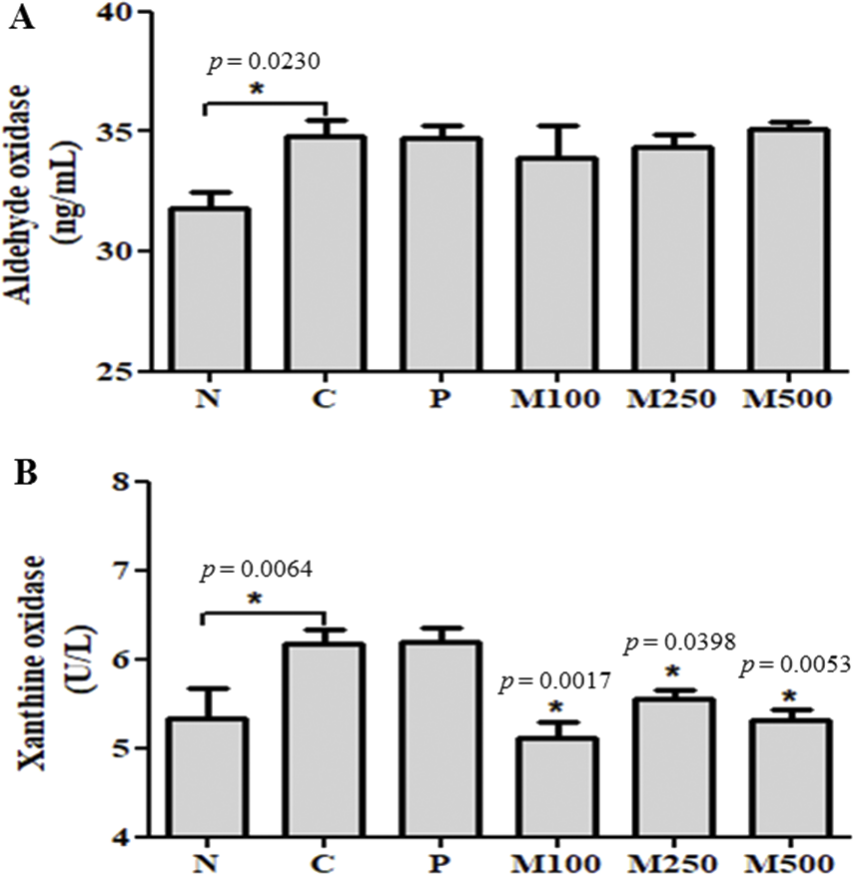
Effect of mackerel muscle protein hydrolysate on aldehyde oxidase and xanthine oxidase activity in liver. (A) aldehyde oxidase and (B) xanthine oxidase activityValues are expressed as mean ± S.E. for groups of eight animals; N: normal; C: control; P: positive control; M100, M250, and M500: mackerel muscle protein hydrolysate at concentrations of 100, 250, and 500 mg kg^−1^, respectively. n.s: not significant; ∗*p* < 0.05 vs. control, †*p* < 0.001 vs. control *p: p* value of ANOVA and significance between control and test groups using Fisher’s PLSD post hoc test.

AO activity was 34.82 ± 1.34 U L^−1^ in group C fed the Lieber-DeCarli ethanol diet, which was significantly (*p* < 0.05) different than the activity in group N (31.83 ± 1.34 U L^−1^; Fig. 4A). AO activity in the M100 group was 33.91 ± 3.08 U L^−1^, which was similar to the level of activity in group P, but different from the level of XO enzyme activity.

XO activity in group C fed the Lieber-DeCarli ethanol diet was 6.17 ± 0.35 U L^−1^, which was significantly higher (*p* < 0.05) than the activity seen in group N (5.33  ± 0.78 U L^−1^; Fig. 4B). XO activity levels in the MMPH sample groups (M100, M250, and M500) were significantly (*p* < 0.05) lower than the activity in group C. Group M100 showed the lowest XO activity of 33.91  ± 3.08 U L^−1^.

### SOD, CAT activity and protein expression

**Figure 5:**
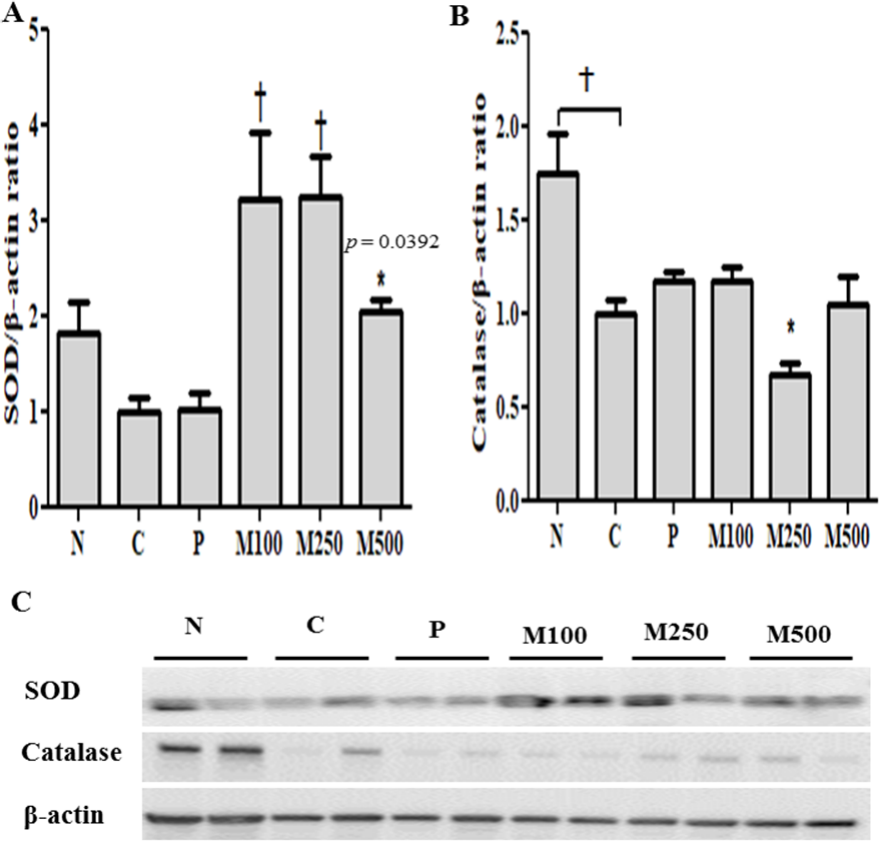
Effect of mackerel muscle protein hydrolysate on SOD and CAT protein levels in liver. (A) SOD and (B) CAT protein levels; (C) SOD and CAT protein expression measured by western blotValues are expressed as mean  ± S.E. for groups of eight animals; N: normal; C: control; P: positive control; M100, M250, and M500: mackerel muscle protein hydrolysate at concentrations of 100, 250, and 500 mg kg^−1^, respectively.Protein extracts were separated on 12% SDS-polyacrylamide gels and after transfer to polyvinylidene fluoride membranes, proteins were detected with monoclonal SOD and CAT antibodies (Santa Cruz Biotechnology) and subsequently, visualized with a goat anti-mouse IgG (H+L) HRP-conjugated secondary antibody (Thermo Fisher Scientific). SOD and CAT expression were detected using a West Save Gold western blot detection kit (Abfrontier), and band intensity was measured using a Chemi-Doc XRS system (Bio-Rad). Results were normalized using a β-actin monoclonal antibody (Santa Cruz Biotechnology) as an internal standard. Additionally, experiments were repeated at least three times to minimize errors. n.s: not significant; ∗*p* < 0.05 vs. control, †*p* < 0.001 vs. control. *p: p* value of ANOVA and significance between control and test groups using Fisher’s PLSD post hoc test.

SOD activity was lower in ethanol-fed group C (1.000 ± 0.387 U mL^−1^) compared to that of group N (1.825 ± 0.788 U mL^−1^), whereas, significantly (*p* < 0.05; *p* < 0.001) higher protein levels were observed in all groups administered MMPH for 6 weeks (Fig. 5A). SOD activity was normalized by comparing with *β*-actin. SOD activities of 3.213 ± 1.876, 3.250 ± 1.13, and 2.036 ± 0.402 U mL^−1^, were observed in the M100, M250, and M500 groups, respectively.

CAT activity in group C (1.000 ± 0.214 U mL^−1^) fed the Lieber-DeCarli ethanol diet for 6 weeks was significantly (*p* < 0.001) lower than that of group N (1.745 ±0.599 U mL^−1^). However, oral administration of MMPH, especially at a dose of 100 mg kg^−1^, increased CAT activity to the level of group P (Fig. 5B). CAT activities of 1.165 ± 0.239, 0.674 ± 0.149, and 1.052 ± 0.422 U mL^−1^, were observed in the M100, M250, and M500 groups, respectively. Western blot results showed higher SOD protein expression and relatively sufficient CAT protein expression levels in groups administered MMPH (Fig. 5C), as compared to the controls.

## Discussion

Free radical scavengers such as antioxidants interact with and neutralize free radicals and prevent them from causing cellular injury in biological systems (Atawodi, 2011). Due to technological advancements, relatively low production costs, and simple extraction methods, marine sources have great potential for use in the production of antioxidant protein hydrolysates and peptides. Protein hydrolysates are considered one of the major sources of bioactive peptides. There are several reported methods for the isolation of antioxidant protein hydrolysates from marine sources, including Alaska pollock (Jia et al., 2010), Pacific whiting (Mazorra-Manzano et al., 2012), tilapia (Fan et al., 2012), and yellowfin tuna (Oliveira et al., 2017). However, with the exception of a previous report from our group (Bashir et al., 2018a), there are no reports on the production of muscle protein hydrolysates by the enzymatic hydrolysis of mackerel.

Previously, we reported on the production of *S. japonicus* protein hydrolysates using proteases (Alcalase, Neutrase, and Protamex). Whole muscle protein hydrolysates prepared using Protamex showed higher *in vitro* antioxidant activities than hydrolysates prepared using the other proteases (Bashir et al., 2018a). *In vitro* experimental results in the previous study showed a maximum degree of hydrolysis of 86.78 ± 1.26%, an ABTS-radical scavenging activity of 95.16 ± 1.00%, a DPPH-radical scavenging activity of 71.69 ± 2.56%, and a SOD-like activity of 32.22 ± 1.47% in mackerel protein hydrolysates. Significantly higher (*p* < 0.05) levels of hydrolysis, ABTS- and DPPH-radical-scavenging activities, and SOD-like activity of protein hydrolysates prepared using Protamex, suggested that Protamex has higher substrate affinity and therefore, would be effective at hydrolyzing mackerel muscle in this study.

All experimental groups in this study showed a consistent weight gain at the start of the experiment. However, there were no significant changes in body weight, organ or tissue weight, or relative liver and epididymal white adipose tissue weight with different experimental treatments except in the M100 group, which showed reduced kidney weight compared to the intact control group. Similar to our study, Nam et al. (2011), Bertola et al. (2013), and Donepudi et al. (2018) reported a weight-losing tendency in animals fed the Lieber-DeCarli ethanol diet. Brien et al. (2011) also reported on the effects of sustained ethanol intake on the ethanol oxidation system and ATP production, which led to weight loss. This phenomenon may be due to a nutritional deficiency caused by decreased dietary intake.

The liver uses fatty acids to synthesize triglycerides and it releases free fatty acids into the blood. However, chronic intake of excess ethanol leads to the accumulation of triglycerides in hepatocytes and fats in the liver, thus resulting in a fatty liver disease (Cao et al., 2016). In the present study, a significant (*p* < 0.001) increase in triglyceride and cholesterol concentration was observed in the Lieber-DeCarli-ethanol-fed group. However, a significant (*p* < 0.001) decrease in triglyceride and cholesterol concentrations was observed in the silymarin- and MMPH-treated groups. This suggests that the protein hydrolysates used in this study are directly involved in fat metabolism. This is consistent with Fengler’s study (Fengler et al., 2016), where long-term ethanol administration resulted in increased total cholesterol concentration in the blood, thereby affecting fat metabolism and inducing fatty liver (McClain et al., 2011). In contrast, despite the Lieber-DeCarli ethanol diet, serum ALT and AST activities were within the normal range for mice (ALT: 17-77 U L^−1^, AST: 54-298 U L^−1^) and results were similar to those of a previous study (Fengler et al., 2016).

MDA is known to cause oxidative damage by triggering the peroxidation of lipids, which are a key component of the cell membrane (Ayala, Muñoz & Argüelles, 2014). Chronic alcohol intake inactivates proteins in the mitochondria and cytosol of liver cells by increasing ROS and inhibits the structural and functional activity of the liver (Bailey, 2018). In the present study MMPH administration, especially at the 100 mg kg^−1^ dose, resulted in decreased lipid peroxidation and improvement in fatty liver disease induced by the Lieber-DeCarli ethanol diet. Similar to our study, enzyme hydrolysis of *Mactra veneriformis* resulted in reduced lipid peroxidation levels (Liu et al., 2015). The ability of a hydrolysate to prevent lipid peroxidation depends on its hydrophobicity (Onoja et al., 2014; Zou et al., 2016). Thus, the antioxidant activity of the protein hydrolysate used in this study may be due to its higher hydrophobic amino acid content, which effectively protected lipids from peroxidation. This suggests that the abundant amino acids of mackerel protein hydrolysate may enhance the antioxidant activity of hepatocytes and inhibit lipid peroxidation, thereby lowering oxidative stress caused by alcohol.

AO and XO can induce ROS-related diseases by generating ROS using molecular oxygen as an electron acceptor (Kundu, Velayutham & Zweier, 2012). They are known to be the enzyme systems that produce active oxygen in the cytosol of liver cells. XO catalyzes the oxidation of xanthine to uric acid using a hydrogen (electron) acceptor (Kostić et al., 2015). Adiponectin, which is usually an anti-obesity adipokine, increases the activity of AO (Pryde et al., 2010), and it is involved in the response to a high-fat diet and the development of fatty liver disease due to ethanol intake (Gamberi et al., 2018). AO and XO activities in groups administered MMPH for 6 weeks, were significantly (*p* < 0.05) lower than the Lieber-DeCarli ethanol diet group, which shows that there was minimal production of active oxygen radicals in MMPH-administered groups. Therefore, the abundant amino acids present in MMPH appear to act in the cytosol of liver cells to regulate ROS, XO, and consequently, lipid peroxidation.

SOD is an enzyme primarily involved in the cell’s defense against oxidative damage, by converting the superoxide anion-radical into H_2_O_2_ (Ighodaro & Akinloye, 2018). H_2_O_2_ is then converted to water by CAT or glutathione peroxide-mediated glutathione and is released from the body (Lubos, Loscalzo & Handy, 2011). When animals are chronically fed ethanol, SOD activity decreases. This is because reactive oxygen species, including NADH and the superoxide anion, are produced due to the large amount of alcohol dehydrogenase, which inhibits SOD protein expression and interferes with the detoxification of active oxygen in the body (Lobo et al., 2010). CAT, an enzyme of the active oxygen reduction system, like SOD, is also reported to decrease its activity in animals with chronic ethanol consumption (Reddy et al., 2014). In the present study, significantly (*p* < 0.001) reduced SOD and CAT activities were observed in the ethanol-fed group, compared with the intact control group. However, the oral administration of MMPH resulted in significantly (*p* < 0.001) higher SOD activity and CAT activity regulated to the level of the silymarin-treated group. Interestingly, higher SOD expression and relatively controlled CAT protein expression levels were seen in all groups administered MMPH. This agrees with the previously reported findings that SOD activity increases in the presence of antioxidants that regulate active oxygen, such as MMPH (Liu et al., 2015). Kumar, Nazeer & Jaiganesh (2012) and Liu et al. (2015) also reported higher SOD and CAT activities in protein hydrolysates and peptides isolated from different marine animals. In our study, the negative effect caused by excessive free radicals in the negative control group was significantly reversed by the application of the mackerel protein hydrolysate. This may be due to the fact that most of the physiological and functional attributes of the proteins are linked to peptides, which are mostly inactive within the sequences of the parent proteins, but become biologically active after hydrolysis (Nazeer, Kumar & Ganesh, 2012). This suggests that the protein hydrolysates used in this study are not only active oxygen producers, but also play a significant role as detoxification regulators in liver tissue.

## Conclusions

The aim of this study was to determine the antioxidative potential of the *S. japonicus* protein hydrolysates fed to mice with alcoholic fatty liver disease. Oral administration of the protein hydrolysate resulted in decreased and controlled serum triglyceride levels and XO activity. At all tested doses (100, 250, and 500 mg kg^−1^) of the protein hydrolysate, lipid peroxidation levels were adjusted to the level of the group treated with silymarin. This was especially true for the 100 mg kg^−1^ dose. This confirms that the mackerel protein hydrolysate directly participated in the production of active oxygen in liver tissue. The expression of SOD protein, which is involved in the active oxygen detoxification system, was also significantly upregulated in the groups administered MMPH. Furthermore, relatively higher SOD activity was observed after treatment with MMPH at 100 mg kg^−1^. This indicates that the abundant amino acids present in MMPH acted in the cytosol of liver cells and enhanced antioxidant activity by directly participating in the expression of XO and the detoxifying proteins, SOD and CAT. The protein hydrolysate used in this study suppressed alcohol-induced oxidative stress by inhibiting lipid peroxidation. Based on the findings of this study, it was concluded that protein hydrolysate prepared from mackerel may be a strong antioxidant, as it regulated mechanisms involving the glutathione family, which are known to be secondary enzymes during detoxification. Therefore, *S. japonicus* has potential for use in the development of bioactive compounds. Further research should focus on purification, characterization, and scale-up of the peptides responsible for the antioxidant activity.

##  Supplemental Information

10.7717/peerj.6181/supp-1 Figure S1 Changes in SOD, Catalase and *β*-actin expression levels by western blot (A) SOD expression levels; (B) Catalase expression levels; (C) Beta-actin expression levels N: Normal; C: Control; P: Positive control; M100: Mackerel muscle protein hydrolysate at a concentration of 100 mg kg ^−1^; M250: Mackerel muscle protein hydrolysate at a concentration of 250 mg kg ^−1^; M500: Mackerel muscle protein hydrolysate at a concentration of 500 mg kg ^−1^.

10.7717/peerj.6181/supp-2 Supplemental Information 1 Raw data

## Additional Information and Declarations

### Data Availability

The following information was supplied regarding data availability: The primary data has been deposited in the repository of the Institutional Animal Care and Use Committee, Southern Medi-Chem Institute (SEMI), Busan, Republic of Korea (Registration No. SEMI-17-01). It is also available as a Supplemental File.
