# Supplementary material for: In vivo antioxidant activity of mackerel (Scomber japonicus) muscle protein hydrolysate
Source: PeerJ. 2018 Dec 21;6:e6181. doi: 10.7717/peerj.6181 (PMC6305115; doi:10.7717/peerj.6181)
Supplement: Figure S1 — (A) SOD expression levels; (B) Catalase expression levels; (C) Beta-actin expression levels N: Normal; C: Control; P: Positive control; M100: Mackerel muscle protein hydrolysate at a concentration of 100 mg kg −1; M250: Mackerel muscle protein hydrolysate at a concentration of 250 mg kg −1; M500: Mackerel muscle protein hydrolysate at a concentration of 500 mg kg −1. [file peerj-06-6181-s001.pdf]

A

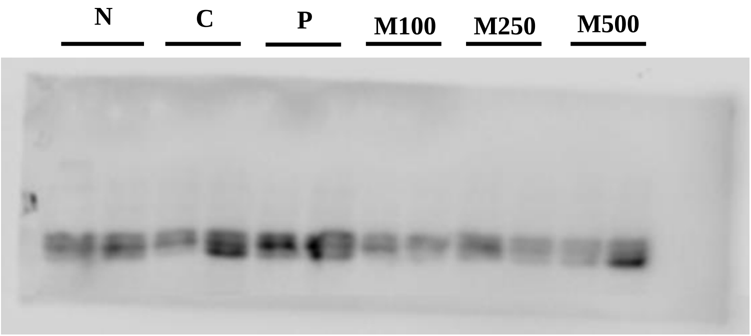

B

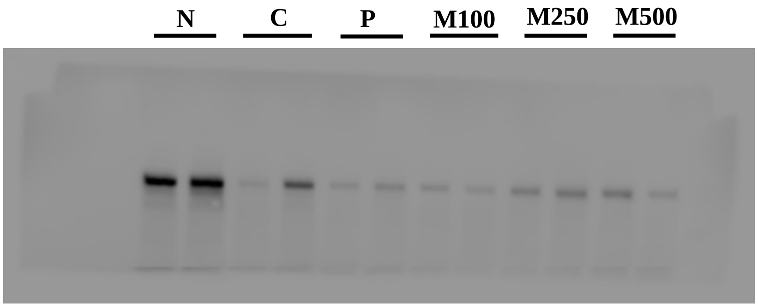

C

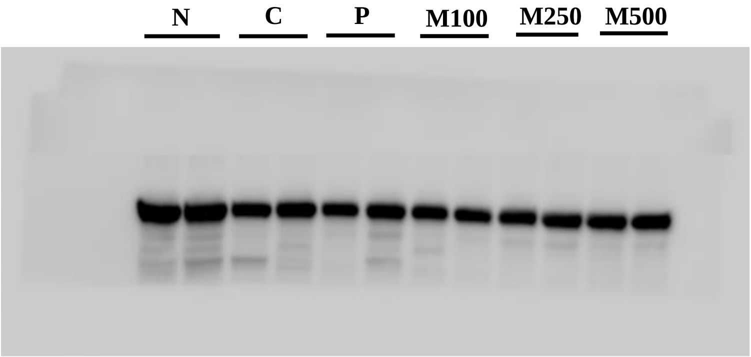

Figure S1: Changes in SOD, Catalase and β-actin expression levels by western blot

A) SOD expression levels; B) Catalase expression levels; C) Beta-actin expression levels

N: Normal; C: Control; P: Positive control; M100: Mackerel muscle protein hydrolysate at a concentration of 100 mg kg^{-1}; M250: Mackerel muscle protein hydrolysate at a concentration of 250 mg kg^{-1}; M500: Mackerel muscle protein hydrolysate at a concentration of 500 mg kg^{-1}.
